# Bright environmental light improves the sleepiness of nightshift ICU nurses

**DOI:** 10.1186/s13054-018-2233-4

**Published:** 2018-11-13

**Authors:** John E. Griepentrog, Hanna E. Labiner, Scott R. Gunn, Matthew R. Rosengart

**Affiliations:** 1 Department of Surgery, University of Pittsburgh, 200 Lothrop Street – Suite F1266.1, Pittsburgh, PA 15213 USA; 2 Department of Critical Care Medicine, University of Pittsburgh, Pittsburgh, PA USA

**Keywords:** Light, Circadian, Shift work sleep disorder, Night shift, Nurse

## Abstract

**Background:**

Shift work can disturb circadian homeostasis and result in fatigue, excessive sleepiness, and reduced quality of life. Light therapy has been shown to impart positive effects in night shift workers. We sought to determine whether or not prolonged exposure to bright light during a night shift reduces sleepiness and enhances psychomotor performance among ICU nurses.

**Methods:**

This is a single-center randomized, crossover clinical trial at a surgical trauma ICU. ICU nurses working a night shift were exposed to a 10-h period of high illuminance (1500–2000 lx) white light compared to standard ambient fluorescent lighting of the hospital. They then completed the Stanford Sleepiness Scale and the Psychomotor Vigilance Test. The primary and secondary endpoints were analyzed using the paired *t* test. A *p* value <0.05 was considered significant.

**Results:**

A total of 43 matched pairs completed both lighting exposures and were analyzed. When exposed to high illuminance lighting subjects experienced reduced sleepiness scores on the Stanford Sleepiness Scale than when exposed to standard hospital lighting: mean (sem) 2.6 (0.2) vs. 3.0 (0.2), *p* = 0.03. However, they committed more psychomotor errors: 2.3 (0.2) vs. 1.7 (0.2), *p* = 0.03.

**Conclusions:**

A bright lighting environment for ICU nurses working the night shift reduces sleepiness but increases the number of psychomotor errors.

**Trial registration:**

ClinicalTrials.gov, NCT03331822. Retrospectively registered on 6 November 2017.

## Background

Shift work, work that occurs outside a timeframe of approximately 0600–1800 h, causes a disruption of circadian biology and underlies what is called shift work sleep disorder (SWSD) [1]. SWSD is characterized by insomnia, fatigue, and excessive sleepiness, and can lead to reduced work performance, processing errors, accidents, absenteeism, and reduced quality of life [2]. The American Academy of Sleep Medicine estimates the prevalence of SWSD to be 5–8%, with night shift workers being affected the most [3]. The significance is underscored when viewed in the context of industrialized nations, in which shift workers comprise more than 20% of the workforce [4, 5]. For a field like healthcare that functions independent of the “time on the clock”, the prevalence of SWSD is likely much higher [6]. Thus, the ramifications of SWSD for the workforce that enables and maintains 24-h healthcare are likely many and profound.

Night shift work forces individuals to work at a time when the circadian sleep drive is high and to sleep when wakefulness is high. This misalignment induces poor daytime sleep and impaired nighttime alertness [7]. Alertness and overall performance reach a nadir in the early morning, yielding reduced work efficiency and a period of increased vulnerability to the commission of errors [8–10]. Inefficient daytime sleep leads to continued reduced night shift alertness and performance, and a vicious cycle of circadian malalignment ensues. Not surprisingly, several interventions have been studied as measures to counter the degradation in fatigue and foster improved sleep post-shift - bright light, naps, and caffeine.

The paradigm that environmental light can be manipulated to modulate circadian biology, physiology, and human performance is not new and possesses biologic plausibility [11, 12]. Light is the dominant environmental cue entraining circadian rhythms, and the prototypical mediator is melatonin [13]. With the commencement of the solar day, light travels through a nonvisual optic pathway to suppress melatonin production and bring on wakefulness and alertness [14]. The reverse occurs upon darkness. Shiftwork perturbs circadian homeostasis, and considerable research has focused upon light therapy, circadian biology, and health [5, 15–17]. Bright light even of short duration has been shown to impart acute positive effects in night shift working nurses, including heightened subjective alertness and reduced insomnia, anxiety, and depression [18]. However, the ramifications of light therapy on nursing performance remain to be completely defined [19]. Herein we conducted a randomized crossover clinical trial to address the hypothesis that exposure to bright light reduces post-shift sleepiness and enhances vigilance and psychomotor performance among ICU nurses working a night shift.

## Methods

### Study design

**Fig. 1:**
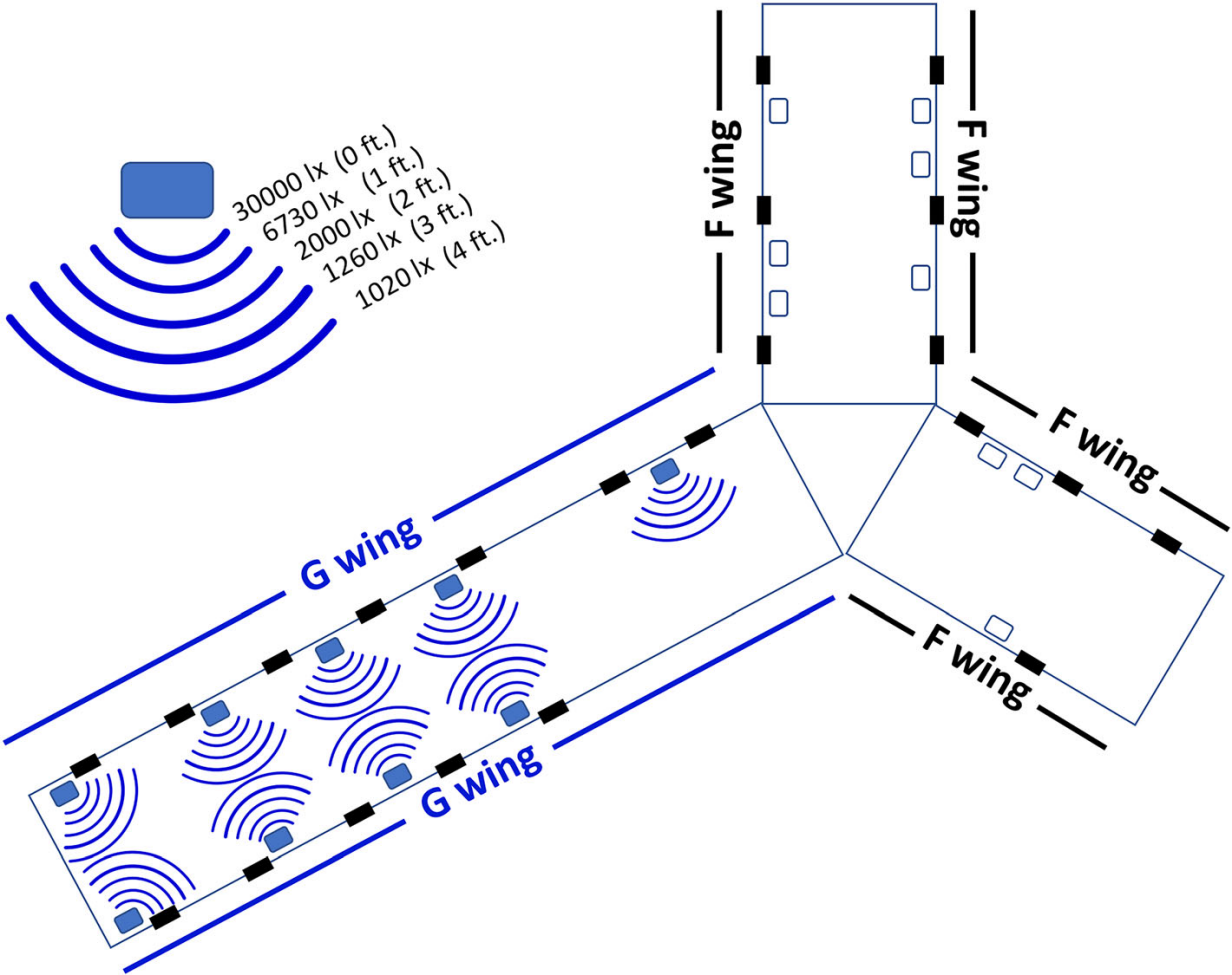
Map of illuminance topography

This is a randomized, crossover clinical trial (NCT03331822) to address the hypothesis that high illuminance light improves vigilance and psychomotor speed while reducing sleepiness among ICU nurses working a night shift. There are two wings to the ICU (F wing and G wing), each with a different geography and perceived potential for varying acuity and magnitude of ICU care (Fig. 1). Thus, we conducted the trial in two phases so as to administer the intervention and the control within each of the two ICU wings. An initial phase was conducted in which the G wing served as the bright light interventional arm and the F wing delivered the standard light and served as the control arm. Upon completion of this phase, a crossover trial commenced in which the conditions were flipped to the other ICU wing. Thus, for the entire trial, both ICU wings produced both light settings.

### Subjects

Eligible subjects were nurses working a night shift (1900–0700 h) in the Surgical Trauma Intensive Care Unit (ICU) at the University of Pittsburgh Medical Center (UPMC) Presbyterian Hospital. Trial enrollment occurred between 1 October and 1 December 2017. This study was approved by the Institutional Review Board (IRB) at the University of Pittsburgh (PRO#17010109). Subjects were enrolled after written informed consent was obtained.

### Baseline characteristics

For each subject we recorded baseline demographics and pertinent characteristics. Visual acuity was self-reported and typically based upon outpatient optometry/ophthalmology visits. The use of over-the-counter and prescription sleep aids was self-reported.

### Randomization

Subjects were assigned in a crossover design to be exposed to both the control and the intervention during each phase of the study. Specifically, for each night shift, nurses were assigned to one of two wings of the ICU (F side or G side) that each had been fitted to provide a distinct lighting exposure: (1) continuous high illuminance white light or (2) the standard, ambient white, fluorescent hospital lighting (Fig. 1). Allocation to either exposure was dictated by the monthly ICU nursing schedule, an established process to staff the ICU that has been performed for years and that occurred independent of our trial design and implementation. Specifically, the nursing assignments to fully staff the ICU are generated well in advance in month-long blocks. For each night, a nurse is assigned to paired beds within one wing of the ICU. Thus, their assignment to either control or intervention was based upon nursing bed assignment. After 4 weeks the lighting exposure layout was flipped and allocation to either intervention or control was again dictated by the monthly ICU nursing assignment. Subjects completing night shifts on both the F and G wings, and thus receiving both exposures, served as matched pairs for analysis.

### Intervention

Before randomization each subject was exposed to the ambient white, fluorescent hospital lighting (emission 300 lux (lx), color temperature 3500–4100 K, 100% UV filtration). The “standard” lighting group continued to be exposed to this ambient white, fluorescent lighting for the duration of each 12-h night shift (1900–0700 h, on 2 October 2017: circadian time CT 1142–2341 (circadian day 0719–1900 h)). The “high” illuminance intervention group was additionally exposed to a Day*Light Classic Light (emission 10,000 lx, color temperature 4000 K, 99.3% UV filtration). The light was positioned outside the ICU room at each nursing work station and directed away from the patient bed, thereby shielding the patients from the experimental conditions. Illuminance was maximal at the work station and linearly degraded with increasing distance (Fig. 1). The density and geographic distribution of lighted stations generated a minimal environmental illuminance exceeding 1000 lx for the entire lighted ICU wing. This configuration exposed each nurse positioned within 24 in. of a work station to an illuminance of 1500–2000 lx (Fig. 1). This high illuminance intervention was initiated at 1900 h and discontinued at 0500 h, generating a 10-h exposure period. For the remaining 2 h of the shift (0500–0700 h) the high illuminance cohort was exposed to ambient white, fluorescent lighting as previously described. Though we did not quantify the lighting exposure of each subject, nurses typically spend the majority of the shift within their assigned ICU wing. Nurses assigned to one wing and exposed to one intervention group were geographically separated from the other group.

### Outcomes

Our primary outcomes were the Stanford Sleepiness Scale (SSS) and the number of lapses and errors on the Psychomotor Vigilance Test (PVT) [20]. The standard night shift occurs during the interval 1900–0700 h. We perceived that times nearing the conclusion of the shift comprise a period of potentially increased vulnerability, insofar as reduced vigilance and performance errors may be concerned. Thus, we chose a distinct time of 0500 h, which is 2 h prior to the end of the shift to administer the tests, and all participants were administered the tests at this same time. This time coincides with the immediate termination of the intervention, and thus there is no gap from exposure to measurement, which should minimize bias. Secondary outcomes included median response time domains of the PVT and salivary melatonin concentration.

#### Psychomotor Vigilance Task (PVT)

Each subject completed the PVT 2 h prior to the conclusion of the night shift (0500 h). The PVT is a validated and sensitive test that assesses the cognitive domains of vigilant attention and psychomotor speed as quantitative parameters of partial and total sleep deprivation [21]. It has been employed as a marker of attention deficit in hundreds of studies to date [21, 22]. It is a 3-min test in which the subjects look at a small rectangular screen and press the mouse every time numbers appear. The number of lapses (responses greater than 500 ms) is recorded. For the PVT, participants must maintain vigilant attention on a target box, respond as quickly as possible to the appearance of a stimulus, and avoid responding prematurely. The stimulus is a millisecond timer, and the reaction time in milliseconds is shown as feedback after each response. A total of 28 metric data fields are generated [21]. The “aggregate score” is a calculated metric of the PVT that penalizes based on the percentage of responses that were lapses and the percentage of responses that were early response errors. It is quantified with the following formula:

$$ \mathrm{Aggregate}\ \mathrm{score}={\left(1-\left(\mathrm{Lapses}/\mathrm{Responses}\right)-\left(\mathrm{Errors}/\mathrm{Responses}\right)\right)}^{\ast }\ 100 $$

The “efficiency” score is a summary estimate that deducts for false starts and long responses. An “error” is recorded by either a false start or a coincident false start, and the parameter “errors” is the sum of total errors (i.e., false starts and coincident false starts).

#### Melatonin concentration

Saliva samples were collected from each subject at the start and conclusion of each shift (1900 and 0700 h) using a commercially available kit (Sarstedt Salivette Cotton Swabs 50–809-199, Fischer Scientific, Hampton, NH, USA) [23]. Samples were immediately snap frozen and stored at − 80 °C for future analysis of melatonin concentrations. Sample melatonin concentrations were assayed using a commercially available enzyme-linked immunosorbent assay (ELISA) for human melatonin (LS Bio Human Melatonin ELISA (competitive EIA)- LS-F39279–1, LifeSpan Biosciences Inc., Seattle, WA, USA).

#### Stanford Sleepiness Scale

Subjects completed the SSS 2 h prior to the end of each shift (0500 h). The SSS is a simple 7-point rating scale developed by Hoddes et al. [24] It has become the validated, gold standard for measuring subjective sleepiness levels and has been used in over 100 clinical trials [25, 26].

### Statistical analyses

All analyses followed the intention-to-treat principle. The study was powered to detect a 1-point change in the SSS scores between lighting assignments. A 1-point difference was determined by consensus among the investigators as a feasible and relevant endpoint. There is no established minimal clinically important difference for this endpoint, though this magnitude of change is in accordance with other studies assessing sleepiness in the context of day and night clinical duties [25]. Assuming a mean SSS score of 2.7 (standard deviation (SD) 1.1), β of 0.80, and two-sided α of 0.05, an estimated 12 matched pairs were needed. Each of the two phases of the trial had a planned enrollment of 20 matched subjects. Thus, we anticipated enrolling approximately 40 matched pairs, which would provide 80% power to detect a statistically significant difference in effect size of 0.50 in SSS scores. We did not interfere with the nursing assignments, and thus each nurse may work several nights assigned to either the intervention or control arms. In these circumstances in which a subject could potentially contribute multiple results for an exposure (e.g., assigned to several night shifts over the study period), we calculated and analyzed the mean subject parameter scores for each lighting exposure (i.e., ICU wing). Thus, for each participating nurse only one pair of matched data were analyzed for each phase of the trial: (1) a single data set for exposure to the bright light intervention and (2) a single data set for the exposure to the control standard light arm. The primary and secondary endpoints were analyzed using the paired *t* test. Data were analyzed using STATA SE 14.

## Results

**Fig. 2:**
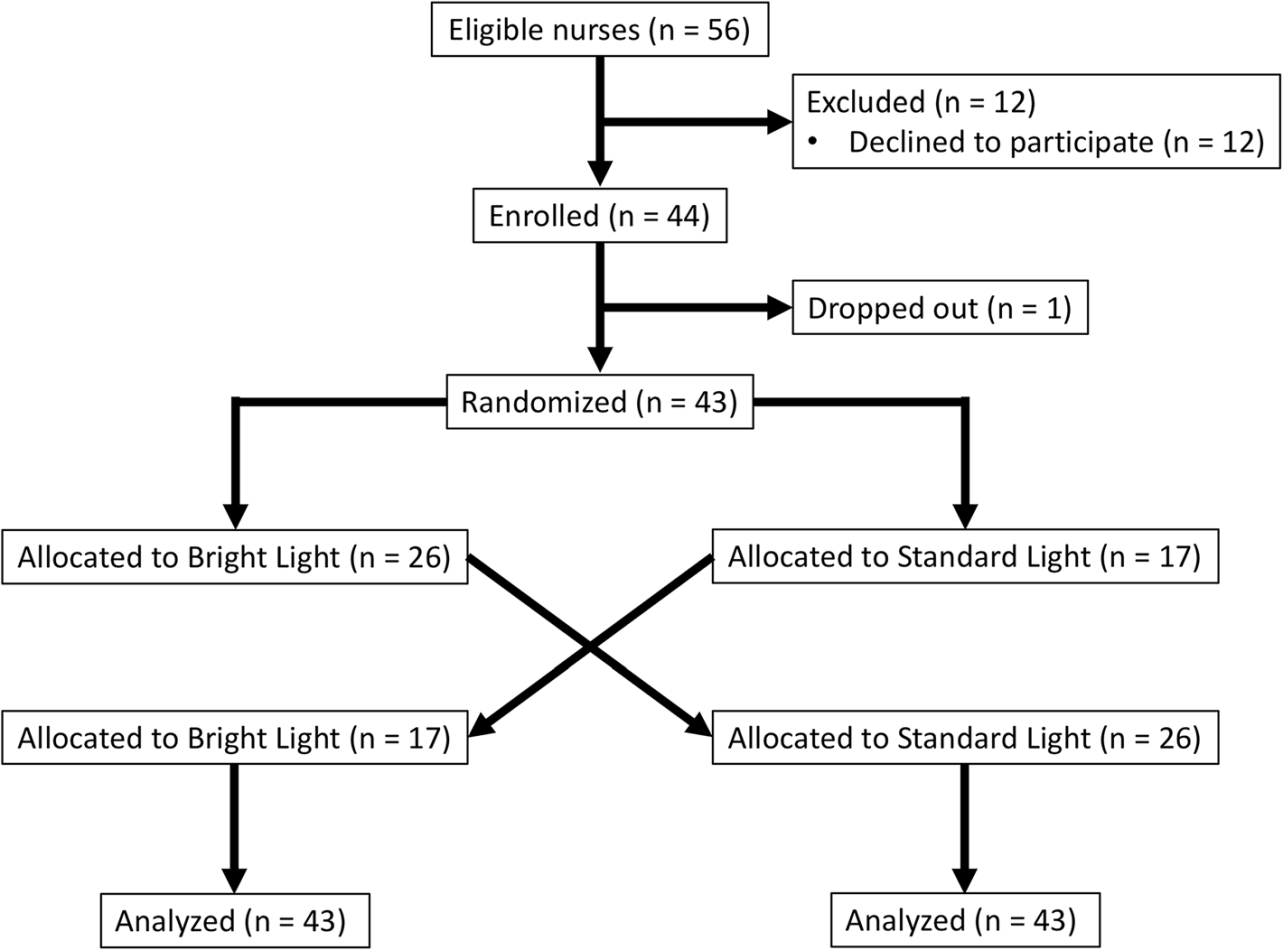
Consolidated Standards of Reporting Trials (CONSORT) diagram

A total of 31 subjects were randomized and completed both lighting exposures. Twelve subjects were enrolled in both phases of the trial, and thus a total of 43 matched pairs comprised the entire cohort for analysis of primary and secondary outcomes (Fig. 2). The median (IQR) age was 29 years (IQR, 26–32 years) and 22 (71%) were female. The median corrected visual acuity of the entire cohort was 20/20 (IQR, 20/20–20/30). Five subjects (16%) utilized nightly melatonin, and five (16%) utilized additional sleep aids.

**Fig. 3:**
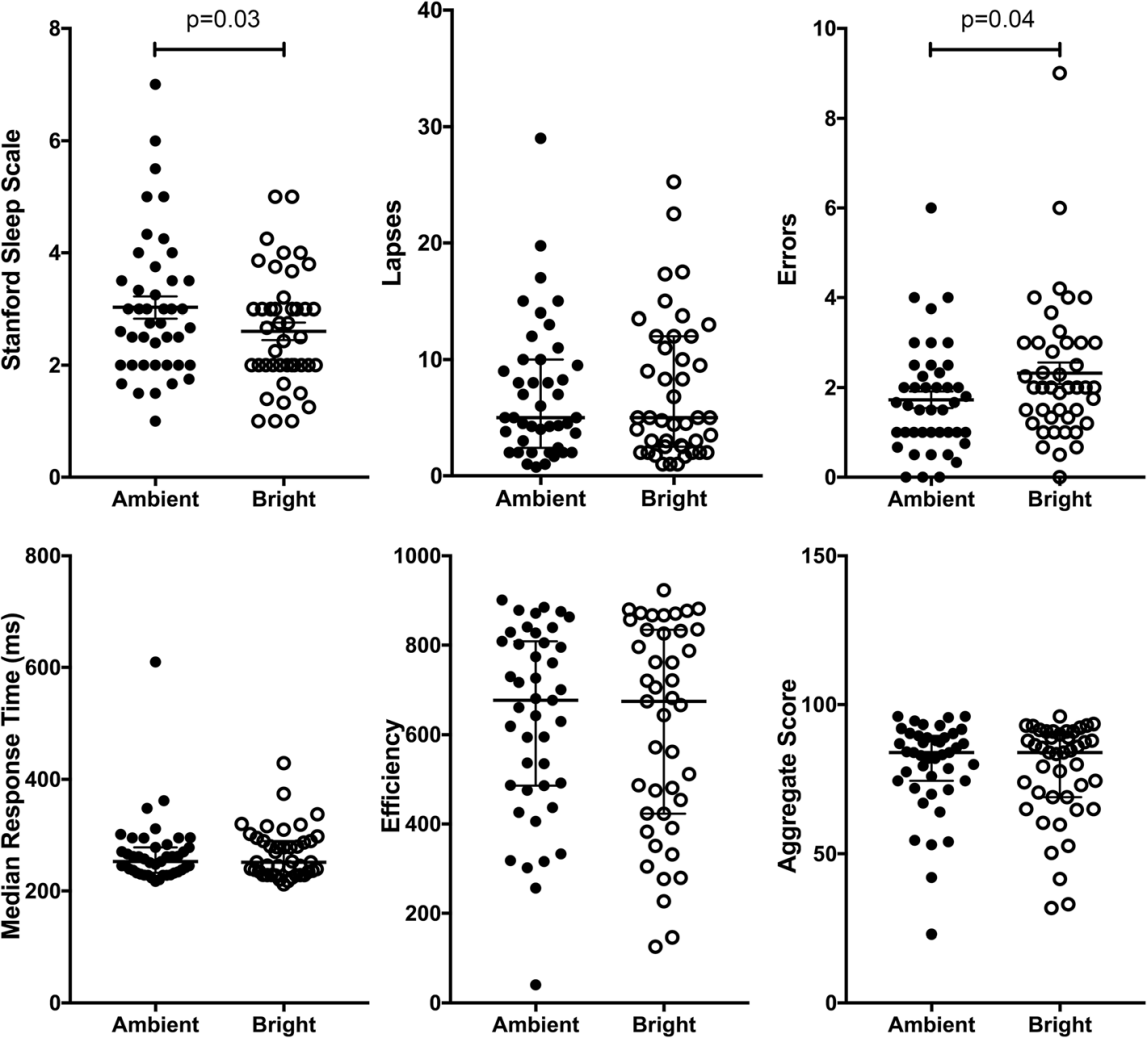
High illuminance lighting reduces sleepiness and increases psychomotor errors

Exposure to high illuminance lighting relative to standard hospital lighting significantly reduced subjective sleepiness at the end of the night shift as quantified by the SSS: mean (sem) SSS 2.6 (0.2) vs. 3.0 (0.2), mean difference − 0.4 (0.2), *p* = 0.03 (Fig. 3). However, after exposure to high illuminance lighting, subjects committed more errors: mean (sem) 2.3 (0.2) vs. 1.7 (0.2), mean difference, 0.6 (0.3), *p* = 0.03 than after exposure to standard hospital lighting. There were no significant differences in number of lapses or in mean or median response times (Fig. 3).

**Fig. 4:**
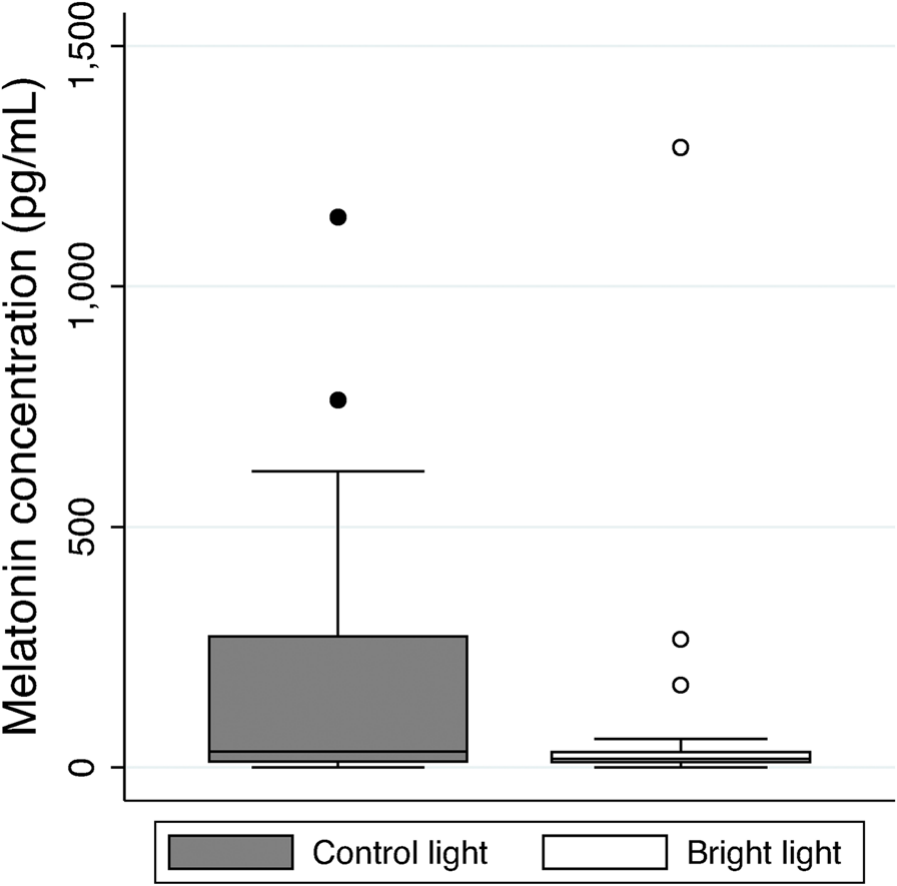
Salivary melatonin concentrations

A total of eight subjects completed paired salivary sample acquisition. Exposure to bright light suppressed early morning salivary melatonin concentrations to a greater degree than standard lighting, though this was not statistically significant: 15 (4.9) pg/mL vs. 44 (32) pg/mL, mean difference − 29 (32), *p* = 0.39 (Fig. 4).

## Discussion

The number of shift workers in industrialized nations is increasing to over 20% of the workforce [5]. In most intensive care units, nurses work 12-h shifts that consist of days and nights. Shift work has been reported to cause fatigue, induce sleep disorders, and cause metabolic disturbances. Exposure to bright light has been shown to improve the subjective symptoms of night shift work, such as sleepiness and vigor [5, 27]. Here we observed that prolonged exposure to high-intensity lighting during the night reduced post-shift subjective sleepiness among ICU nurses but resulted in more errors; there was no significant effect on other parameters of the PVT such as lapses and response times. Thus, although continuous bright light during the scotophase may contribute to improving nursing wakefulness, it does not appear to improve performance, and our data suggest it may worsen it.

Alertness and overall performance in diurnal creatures are degraded as the early morning approaches, increasing the susceptibility to committing errors and reducing work efficiency [8–10]. This fact provided the impetus for us to assess PVT and SSS parameters at 0500 h. Several interventions have been studied as measures to counter the degradation causing fatigue, though light, the predominant cue entraining circadian biology and sleep/wake cycles, has been the focus of particularly intense investigation [11, 12]. Exposing human subjects during the early biological night to either 106 or 9100 lx, relative to 3 lx, has been shown to acutely suppress plasma melatonin, advance the time of the circadian pacemaker, and increase alertness, as assessed by the Karolinskia Sleepiness Scale (KSS) and Karolinska Drowsiness Test (KDT) [28]. Additional studies have had similar results, with reduced sleepiness and fatigue independent of whether the exposure to high (i.e., 5000 lx) illuminance occurred during the day or night [29, 30]. These data support that varying brightness even within the typical range of ambient lighting can significantly improve subject alertness. However, the control conditions utilized by these studies approximated complete darkness and are distinct from the lighting typical of an ICU (i.e., 100 and 300 lx). Thus, heightening alertness and performance in this latter hospital environment may prove more challenging.

In this vein, contemporary studies have shown that hospital night shift workers exposed to either short photoperiods of high intensity light or dynamic lighting that replicated daylight variations felt more alert and well-rested after sleep [5, 16]. In a study of 113 ICU nurses, subjects exposed to dynamic lighting that replicated natural daylight variations experienced more effective sleep; however, this study did not assess parameters of alertness or vigilance during the actual night shift [5]. In a simulated shift work protocol, exposure to modestly bright (> 2000 lx) white light at night improved alertness and attention, preserved sleep duration following the night shift, reduced response times and lapses on the PVT, and advanced circadian timing relative to the control group exposed to ambient (100 lx) lighting [31]. These later results are compelling, though the simulated laboratory conditions render them difficult to generalize to the actual workplace. In a more “real world” assessment of the ramifications of altering light within a hospital setting, a 10-min exposure to bright (5000 lx at 40 cm) light prior to the day shift (0800–1700 h) reduced sleepiness and reduced mean response time on the PVT at 1000 h, though no significant difference was observed in any parameter later in the day [32]. A more recent study of ICU nurses reported that cognitive performance, self-reported depressive signs and symptoms, and fatigue did not differ significantly between exposure to 1700 lx during the day time versus 300 lx in control settings [19]. These data suggest that a higher illuminance environment may prove beneficial to both the day and night shift worker, but that there is likely a lower threshold of light illuminance and of exposure duration.

Our trial was one of effectiveness and evaluated the effects of bright white light on ICU nurse vigilance during a working night shift. Though sleepiness towards the conclusion of the shift was reduced, high illuminance failed to beneficially effect several parameters of the PVT, such as mean response times and lapses. Notably, and certainly relevant to the delivery of healthcare, there was an increase in errors after prolonged exposure to bright white light. Other investigators have similarly reported that altering the night lighting environment may yield counter-intuitive cognitive or behavioral results. In a study of simulated driving at early night, blue-enriched white light enhanced physiological arousal but did not correlate with improved cognition; indeed, blue light produced larger driving errors than amber light [33]. The authors concluded that excessive arousal might deteriorate accuracy in complex tasks requiring precision, in this case driving [33]. We similarly consider this mechanism as a plausible underlying cause of our paradoxical results.

Emerging data suggest that a shorter spectrum, such as blue light, may have a greater impact on modulating circadian rhythms and heightening alertness [15]. Melanopsin, the circadian photopigment of the eye has a peak absorption of 467 nm, and thus, it is light of the lower wavelength, visible blue spectrum that maximally entrains circadian biological processes [34–36]. Several studies have evaluated whether blue light more effectively enhances alertness and performance [37]. In a before-after intervention study of 30 night-shift workers, blue-enriched white light improved alertness and decreased omission errors and reaction times, markers of cognitive performance [38]. Low illuminance white light rich in the blue spectrum improved subjective sleepiness in night shift workers when it was applied at night between 2300 and 0700 h) [39]. However, the effects of blue-enriched light may be context dependent; in particular, dependent upon the complexity of task performance [33].

One suggested mechanism by which light enhances alertness is through suppression of nocturnal melatonin, the biological mediator of sleep [13]. Several studies have evaluated the suppression of melatonin by phototherapy [39–41], and tested correlation between the physiologic effects of light, including reduced sleepiness and task performance improvement, and reduced melatonin concentration [42]. We too observed reduced melatonin with our intervention of high illuminance, though this was not statistically significant. Unfortunately, salivary samples were not collected from every nurse, which likely limited our power. Alternatively, our study used a much longer photoperiod of high illuminance light, and the mechanism of melatonin suppression could have been exhausted towards the end of shift. Nonetheless, caution must be exercised, as long-term shift work causing reduced melatonin levels has been linked to an elevated risk of a variety of diseases, including cancer [43].

Night shifts force individuals to work at a time when the circadian drive to sleep is high and then to sleep when wakefulness is high. This misalignment induces poor daytime sleep and impaired nighttime alertness [7]. Thus, the ideal intervention will not only improve nocturnal alertness and performance during the shift, but also encourage a rapid onset of sleep, post-shift. In the context of altering the environment, this optimal intervention has yet to be identified. However, the evidence suggests that the period of transition to the day (i.e., the morning) is equally important, insofar as achieving the objective of improved daytime sleep. In a study of bright light exposure at night, a second cohort underwent bright light exposure followed by light attenuation in the morning with the simple intervention of sunglasses. Both groups exhibited improved nocturnal alertness, but only the bright light/sunglasses group experienced improved post-shift daytime sleep. These data suggest that nocturnal alertness and daytime sleep in night shift workers is improved with bright light exposure in their work place, but maximized by attenuating light the following morning prior to daytime sleep [44]. Others have reported similar observations [45, 46]. Even more recent data suggest that a “one size fits all” approach is insufficient, and that a personalized approach accounting for the individual’s baseline circadian phase is needed [47]. Additional studies propose a biological rationale for an effect on sleep of a differential response to light [48].

## Conclusion

In conclusion, we observed that altering the lighting environment of ICU nurses working the night shift to bright light reduced their sleepiness but led to an increased number of errors. As the need for healthcare is independent of the time on the clock, so too the demand for night shift workers is likely to expand. Further studies are required to identify the optimal illuminance, photoperiod and wavelength of light that will best foster circadian realignment, and thereby enhance nighttime vigilance and performance, improve daytime sleep efficiency, and ultimately improve the wellbeing of our night shift nurses and their delivery of care.

## Abbreviations

ICU: Intensive care unit
IRB: Institutional Review Board
lx: Lux
PVT: Psychomotor vigilance task
SSS: Stanford sleepiness scale
SWSD: Shift work sleep disorder
